# *Philodendron luisae* (Araceae), a new species from Rio de Janeiro State, Brazil

**DOI:** 10.1186/s40529-015-0082-x

**Published:** 2015-01-23

**Authors:** Luana Silva Braucks Calazans, Nerivaldo Gomes Antas, Cassia Mônica Sakuragui

**Affiliations:** 1 Instituto de Biologia, Departamento de Botânica, Universidade Federal do Rio de Janeiro, CCS, Av. Carlos Chagas Filho, 373 - Sala A1-088 - Bloco A, Ilha do Fundão, Rio de Janeiro CEP, 21941-902 RJ Brazil; 2 Fundação Luisa Sartori, Sartori Árvores Nativas e Reflorestamento, Fazenda Além do Horizonte Rodovia RJ-126, km 28, s/n – Gaviões, Silva Jardim, Rio de Janeiro, RJ Brazil

**Keywords:** Aroids, Taxonomy, Conservation, Atlantic forest, Silva Jardim

## Abstract

**Background:**

*Philodendron* is the second largest genus of Araceae, being highly diverse in the Atlantic Forest biome, with nearly one third of the Brazilian species occurring in Southern Brazil, particularly in Rio de Janeiro state. During a local inventory in Silva Jardim municipality, we found a peculiar population of *Philodendron* growing in lowland rainforest.

**Results:**

After morphological analysis and comparisons with similar species, the population proved to be a new undescribed species of subgenus *Philodendron* section *Macrobelium*.

**Conclusions:**

The new species, named *Philodendron luisae*, is here described, illustrated and compared to morphologically close species.

**Electronic supplementary material:**

The online version of this article (doi:10.1186/s40529-015-0082-x) contains supplementary material, which is available to authorized users.

## Background

*Philodendron* Schott is the second largest genus of Araceae, with ca. 480 exclusively Neotropical species (Boyce and Croat [2014]). The genus is highly diverse in tropical rainforests, such as the Atlantic Forest of coastal Brazil. Despite the elevated levels of deforestation in the Atlantic Forest (Fundação SOS Mata Atlântica – Instituto Nacional de Pesquisas Espaciais [2014]), this biome accounts for ca. 12% of the genus diversity in its overall range and 40% of the genus diversity in Brazil (Sakuragui et al. [2014]). These numbers are being constantly increased by the recognition of new species, especially from Southeastern Brazil (e.g. Buturi et al. [2014], Calazans and Sakuragui [2013], Coelho [2010], Gonçalves [2011]).

In Rio de Janeiro state, Southern Brazil, the genus is represented by an impressive number of species, despite the reduced geographical range and high levels of habitat fragmentation (Sakuragui et al. [2011]). Of the 168 Brazilian species, 30 occur in the Rio de Janeiro state (Sakuragui et al. [2014]). Although efforts to catalogue the regional flora dates back to the eighteen century, many localities remain poorly known or even uncollected, especially outside the metropolitan area of the Rio de Janeiro City, justifying intensive sampling efforts.

Here we describe *Philodendron luisae sp. nov.*, an only recently collected and recognized species from Rio de Janeiro State’s lowlands.

## Methods

During a flora inventory conducted in the Atlantic Forest remnants in Silva Jardim municipality, Rio de Janeiro State, we found an indeterminable *Philodendron* species. It was recognized as belonging to subgenus *Philodendron* section *Macrobelium* and analyzed through the two more comprehensive and updated keys of the section (Croat [1997], Sakuragui et al. [2005]). The species was also compared with the type specimens and descriptions of morphologically similar species. The descriptive terminology follows Stearn ([2004]) and Mayo ([1991]). Morphological analyzes of fresh and dry materials were performed with the aid of a stereoscopic microscope. The extent of occurrence and area of occupancy were calculated using the GeoCAT tool (Bachman et al. [2011]).

## Results and discussion

**Figure 1:**
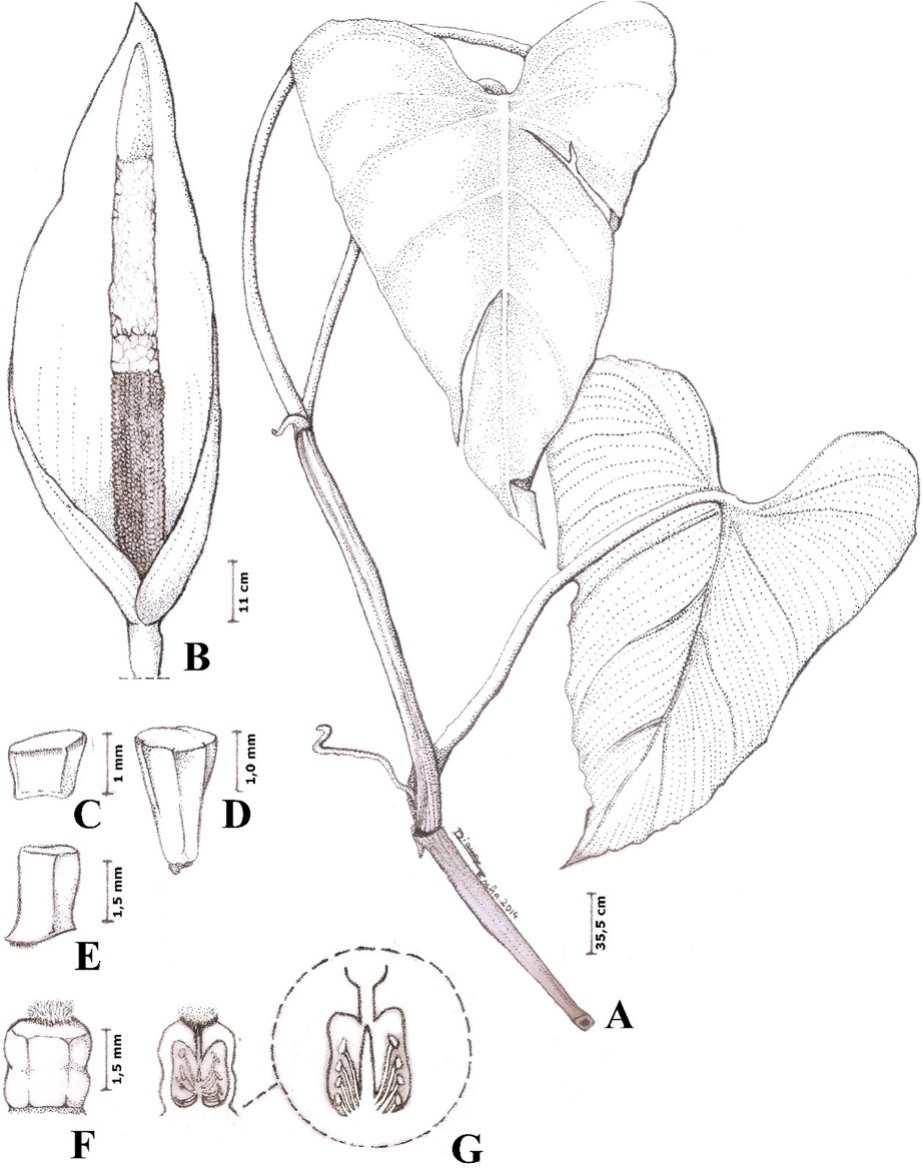
***Philodendron luisae*** **. A**. Habit, x ½. **B**. Inflorescence, x 1. **C.** Apical staminode. **D**. Stamen. **E**. Itermediate staminode. **F**. Gynoecium **G**. Longitudinal cut of gynoecium and the basal placentation (detail). **A** from *Antas et al. 188* (RB); **B-G** from *Antas 181* (RB).

**Figure 2:**
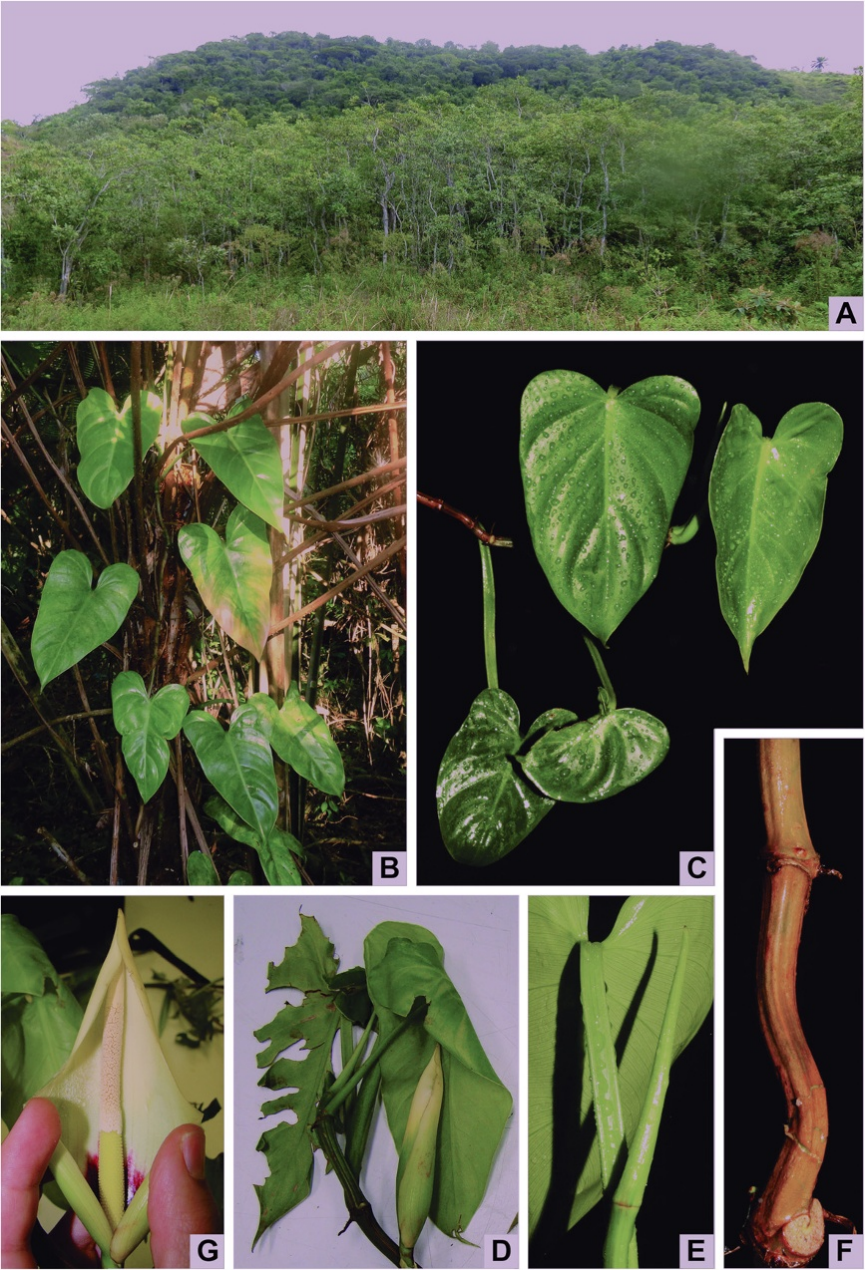
**Habitat and morphology of** ***Philodendron luisae*** **. A**. Habitat in a fragment of Atlantic Ombrophilous Dense Submontane Forest in Silva Jardim municipality. **B**. An hemi-epiphytic individual growing on *Dicksonia* sp. **C**. Young leaves. **D**. Flowering shoot with an inflorescence in pre-anthesis and a leaf ripped. **E**. Detail of the flattened petiole. **F**. Detail of the angular internodes. **G**. Detail of the inflorescence manually opened to show the interior.

*Philodendron luisae* Calazans, *sp. nov.* (Figures 1 and 2).

Type:—BRAZIL. Rio de Janeiro: Silva Jardim, road RJ-126, Sítio Além do Horizonte, 22° 32′ 47.8″ S, 42° 27′ 54.1″ W, 07 November 2013, *N.G. Antas 181* (holotype, RB; isotypes, NY, K).

***Herb*** hemi-epiphytic. ***Internodes*** 4.7–11(−15) cm long, usually shorter in flowering shoots, 3–4–angular, keeled, greenish becoming light brown, drying often cracked with rhytidome-like layers; **intravaginal squamules** up to 3 per node, inconspicuous, deciduous, becoming dark. ***Prophyll*** 6.8–10 *×* 0.1–1.5 cm, triangular, deciduous, slightly keeled, smooth, yellowish becoming cream, drying brown. ***Petiole*** 11–17 *×* 0.4–0.6 cm, adaxially flattened, abaxially rounded, glossy green, slightly striated, drying dark brown; **leaf blade** 16–20 *×* 9–11.3 cm, triangular to cordate-sagittate, smooth, glossy green, abaxially paler, drying membranous, striated, olive-green, strongly discolorous, margin entire, apex acuminate, acumen 1–2 cm long, sometimes curved, base cordate; **anterior division** 12–15.5 cm long, midrib impressed on both faces, drying dark brown, **primary lateral veins** 3–4 pairs, arising from midrib at 70°, 40° and 25–35° angle respectively from the base to the apex, arcuate to margin, impressed on both faces, drying discrete adaxially, dark brown abaxially, **secondary veins** indistinct, parallel to primary veins, numerous, drying evident on both faces, prominent adaxially; **posterior divisions** 3.5–5 cm long, cordate, primary acroscopic veins 2(−3), basal denudation absent. ***Inflorescence*** solitary; **peduncle** 1.52–2.5 cm long, cylindrical; **spathe** 10–11 cm long, ovate, acuminate, acumen ca. 1 cm long, constriction not evident, externally green becoming cream towards the apex, striated, internally cream, reddish at the base, resin canals internally visible; **stipe** absent; **spadix** 8–9 cm long, slender; apical sterile zone 1.3–2.1 cm long, yellowish; fertile male zone 3–3.6 cm long, yellowish; intermediate sterile zone 0.6–0.8 cm long, cream; female zone 3–4.3 cm long, light green; apical staminodes ca. 1 mm long, prismatic; stamens ca. 1 mm long, prismatic; intermediate staminodes ca. 1.5 mm long, prismatic; gynoecium 1.5–2.0 mm long, ovary ca. 1.5 mm long, barrel-shaped, (6)–7–8–locular, 3–4–ovulate, placentation basal, stylar region ca. 0.75 mm long, as wide as the ovary, stigmatic region ca. 0.75 mm long. ***Berries*** unknown. ***Seeds*** unknown.

### Phenology

Collected in flower in November.

### Etymology

The species is named in memory of the Biology undergraduate student Luisa Pinho Sartori, who inspired the conservation and educational initiatives promoted by Sartori family.

### Distribution and ecology

**Figure 3:**
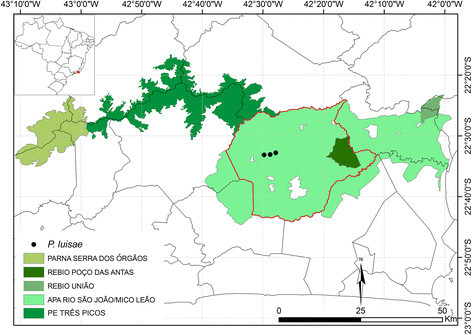
**Distribution map of** ***Philodendron luisae*** **.** The red spot show the focus area in the Rio de Janeiro state. Green areas represent the ecological corridor Mosaico Central Fluminense composed of different conservation unities. Silva Jardim municipality is highlighted by the red line. APA – Área de Proteção Ambiental; PARNA – Parque Nacional; PE – Parque Estadual; REBIO – Reserva Biológica.

Only known from three records in reduced Atlantic Ombrophilous Dense Submontane Forest fragments in Silva Jardim municipality, a rural zone of Rio de Janeiro state (Figure 3). The species can be found in small patches of submontane and seasonally flooded forests in areas of regeneration, growing mainly in primary *Tabebuia* formations associated to the phorophytes *Tabebuia cassinoides* (Lam.) DC. and *Dicksonia* sp.. This is indicative of the species’ tolerance to open habitats, which may be interesting in reforestation projects. The species is also frequently found growing together with *P. nadruzianum* Sakur.

### Conservation

Our data so far indicate the species as *critically endangered* (CR) - B1ab (ii, iii, iv) - following the categories and criteria of International Union for Conservation of Nature ([2012]), with extent of occurrence estimated in 32.2 km^2^. In this category are inserted species facing a high risk of extinction in the wild, with extent of occurrence <100 km^2^ and very fragmented, known from a single location, with continuing decline inferred for the number of individuals and habitat quality.

All records of *P. luisae* up to now are strongly related to stream margins, an area designated as permanently protected by Brazilian federal law in order to preserve the biodiversity and environmental resources in strategic areas. Additionally, the species is only known from particular properties within the Área de Proteção Ambiental da Bacia do Rio São João/ Mico-Leão-Dourado, a conservation unity of sustainable use (Ministério do Meio Ambiente – Instituto Chico Mendes de Conservação da Biodiversidade [2008]). The region suffered historical fragmentation due to selective extraction of timber and farming practices (Carvalho et al. [2006], Guedes-Bruni et al. [2006]), however, the forest remnants are very representative of the Atlantic Ombrophilous Dense Submontane Forest, being of high priority for conservation (Carvalho et al. [2006]). These remnants compose the ecological corridor Mosaico Central Fluminense along with the conservation units Parque Estadual dos Três Picos, Parque Nacional da Serra dos Órgãos, Reserva Biológica Poço das Antas and Reserva Biológica União (Instituto Estadual do Ambiente [2013]) (Figure 3). Probably, populations of *P. luisae* may be found in the lowlands of this ecological corridor, assured within protected areas.

The association between *P. luisae* and the phorophyte *T. cassinoides* is interesting from the conservational viewpoint since this tree is currently threatened and listed in the Red List of the Brazilian Flora (Lohmann et al. [2013]). The tree has suffered an intensive selective extraction due to its high quality timber, used mainly for the manufacturing of shoes (Lohmann et al. [2013]). This activity probably caused impact on the natural populations of *P. luisae* in the *Tabebuia* formation, contributing to the fragmented distribution currently known.

### Paratype

Brazil, Rio de Janeiro: Silva Jardim, road RJ-126, Fazenda Novo Horizonte, 22° 33′ 08.0″ S, 42° 29′ 49.6″ W, 13 November 2013, *N.G. Antas et al. 188* (MBML, RB, SPF).

### Features and affinities

*Philodendron luisae* can be promptly recognized by its small and fragile leaves (often ripped when adult) and 3–4-angular, light brown colored stem. The overall leaf shape and inflorescence with apical sterile zone makes this species close to *P. simonianum* Sakur. and *P. tenuispadix* E.G.Gonç., but it differs by its longer internodes, smaller leaf blade, number of primary lateral veins, number of inflorescences per sympodium, absence of extrafloral nectaries and spathe features (Table 1). When the leaves are still young, *P. luisae* also resembles *P. fragile* Nadruz & Mayo and *P. millerianum* Nadruz & Sakur. by its triangular and fragile leaves without well developed posterior divisions, but the species show a number of differences between them (Table 1).

**Table 1:** **Comparison between** ***P. luisae*** **and closely related species**

Species	Internodes dimension (cm) and shape	Extrafloral nectaries	Leaf dimension (cm)	Primary lateral veins	Inflorescences per sympodium	Apical sterile zone	Spathe color outside, constriction and opening at anthesis	Resin canals inside the spathe
*P. fragile*	1–5, terete	absent	15–37 *×* 9–24	4–5	1(−2)	absent	greenish becoming cream towards the apex, slightly constricted, moderately opened	not visible
***P. luisae***	4.7–11(−15), markedly angular	absent	16–20 *×* 9–11.3	3–4	1	present	green becoming cream towards the apex, not constricted, moderately opened	visible
*P. millerianum*	2.4–8.5, terete	absent	20.2–21.7 *×* 4–11.1	4	1	present	completely white, strongly constricted, slightly opened	not visible
*P. simonianum*	2–4, terete	absent	36–42 *×* 14–20	3–4	3–4	present	completely white, not constricted, reflexed	not visible
*P. tenuispadix*	3–4, terete	present	51–59 *×* 24–27	6–8	1–3	present	completely green, slightly constricted, almost completely opened, but not reflexed	not visible

Information from *P. millerianum*, *P. simonianum* and *P. tenuispadix* according to the original publications (Coelho and Sakuragui [2007], Sakuragui [2001] and Gonçalves [2002], respectively); from *P. fragile* according to Sakuragui et al. ([2005]).

Moreover, among these related species, *P. luisae* is the unique known to grown in swamp forest, a vegetation formation frequently more open and exposed to seasonality than the humid and shaded forests where the most of *Philodendron* species occurs.

## Conclusions

*Philodendron luisae* is a new species easily recognizable and well supported and represents the 31st species record for Rio de Janeiro State. The species, only known from one locality, exemplifies the importance and urgency of local and regional floras to a broaden knowledge of the Brazilian biodiversity.

## Authors’ original submitted files for images

Below are the links to the authors’ original submitted files for images. Authors’ original file for figure 1 Authors’ original file for figure 2 Authors’ original file for figure 3
