# Supplementary figures and images for: Philodendron luisae (Araceae), a new species from Rio de Janeiro State, Brazil
Source: Bot Stud. 2015 Jan 23;56:1. doi: 10.1186/s40529-015-0082-x (PMC5430322; doi:10.1186/s40529-015-0082-x)

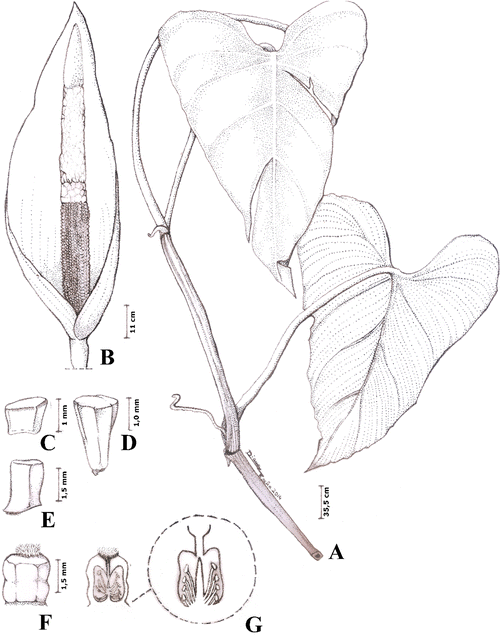

Supplement: Supplementary file 1 — Authors’ original file for figure 1 [file 40529_2015_82_MOESM1_ESM.gif]

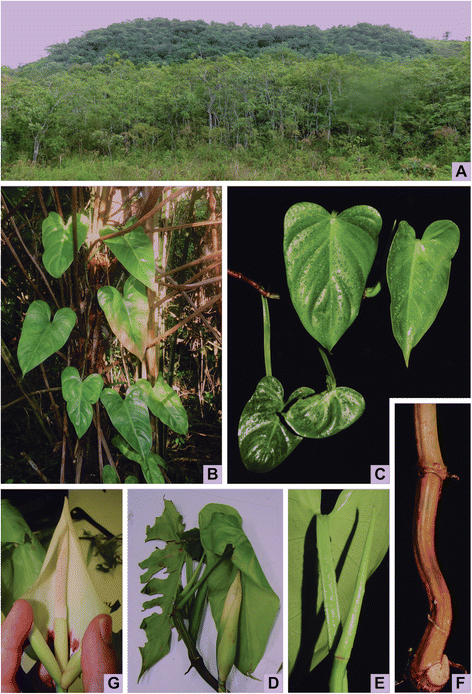

Supplement: Supplementary file 2 — Authors’ original file for figure 2 [file 40529_2015_82_MOESM2_ESM.gif]

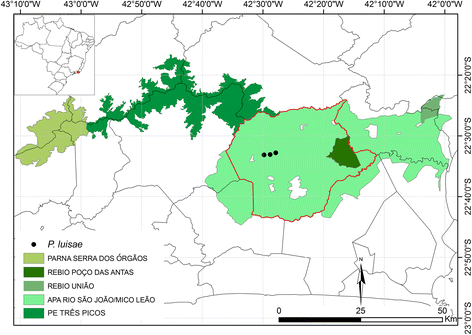

Supplement: Supplementary file 3 — Authors’ original file for figure 3 [file 40529_2015_82_MOESM3_ESM.gif]
